# Precarity, clinical labour and graduation from Ebola clinical research in West Africa

**DOI:** 10.1080/11287462.2019.1566973

**Published:** 2019-01-17

**Authors:** Arsenii Alenichev, Vinh-Kim Nguyen

**Affiliations:** a Department of Anthropology, University of Amsterdam, Amsterdam, Netherlands; b ISGlobal, Barcelona, Spain; c Graduate Institute of International and Development Studies, Geneva, Switzerland

**Keywords:** Ebola, vaccine trial, compensation, reintegration, West Africa

## Abstract

The provision of gifts and payments for healthy volunteer subjects remains an important topic in global health research ethics. This paper provides empirical insights into theoretical debates by documenting participants' perspectives on an Ebola vaccine trial in West Africa. This trial provided hundreds of Africans with regular payments, food packages and certificates for participation. The researchers conducting the trials considered these socioeconomic provisions to be gifts in accordance with contemporary ethical standards and principles. Trial participants viewed them differently, however, approaching trial participation as a means for training and employment in what was from their perspective a new job market: the post-Ebola expansion of research and health care systems. This paper analyses participation in contemporary research by viewing the context-specific histories of trial participants through the lens of prior interventions, specifically participatory reintegration programmes conducted in Anglophone West Africa to overcome civil war crises. In particular, we argue that participation in the Ebola vaccine trial was inadvertently shaped by the design and outcomes of past reintegration programmes. Our results highlight the need to investigate existing socioeconomic landscapes which surround and indeed permeate clinical research as a prerequisite for understanding the participatory motives of vulnerable participants in West Africa and elsewhere.

## Background

In 2014, several clinical trials were launched in West Africa at the peak of the Ebola outbreak, at a time when a nightmare scenario forecast over a million deaths with potentially catastrophic political results (Meltzer et al., 2014). Ebola vaccine and drug trials were hastily implemented whilst international aid agencies and local authorities tried their best to offer care to the Ebola patients. Moreover, Ebola research in Liberia and Sierra Leone was launched in the context of painful recovery from civil war crises, ravaging Sierra Leone between 1991 and 2002, and Liberia between 1989 and 1997, and from 1999 to 2003. These recent conflicts killed hundreds of thousands, with many more displaced and marginalised. Research, aid, and humanitarian agencies scrambled to address overlapping humanitarian and biomedical emergencies. Addressing ethical considerations requires time and careful planning, but the urgency of responding to the epidemic posed difficult and at times unprecedented ethical dilemmas (Caremel, Faye, & Ouedraogo, 2017; Gomez-Temesio & Le Marcis, 2017).

Two specific challenges were reported by participants involved in implementing the trials. On the one hand, due to the public health emergency constituted by the Ebola epidemic, there was a need to deliver trials within the shortest possible timeframe: finding effective vaccines and/or drugs could save countless lives. On the other hand, the burden of research participation had to be minimised for vulnerable participants. Providing participants in trials with payments in instalments, workshops on how to be a trial volunteer, food packages, better health care, awards and certificates for participation represented one solution to these conflicting dilemmas. Those involved in implementing the trials, guided by the principle of minimising risks for vulnerable subjects, saw these socioeconomic measures as ethical gifts aimed to compensate people for their contribution in the midst of the Ebola outbreak. Accordingly, they regarded participation in research to be the result of volunteers' altruistic motives to contribute to science and development, and as an opportunity to receive a potentially life-saving vaccine.

Benefit provision in clinical research has been an active topic of debate among bioethicists, anthropologists and philosophers. In many countries, including those hosting Ebola vaccine research, paying healthy volunteers for clinical research participation is a legal procedure intended as compensation for barriers to enrolment without constituting undue incitement. At the same time, current recommendations and international guidelines, such as those from International Ethical Guidelines for Biomedical Research Involving Human Subjects (CIOMS) and the World Medical Association-Declaration of Helsinki explicitly state that payments for participants must not have persuasive effects on participants’ choices. However, such documents provide minimal guidance on how to calculate or decide whether and how these payments persuade participants (Grady, Dickert, Jawetz, Gensler, & Emanuel, 2005; Pandya & Desai, 2013). Although numerous scholars have proposed various practical solutions, a lack of consensus persists with regards to the ethics of paying healthy volunteers.

It is possible to identify two opposing, broad styles of ethical reasoning concerning the payment of healthy volunteers. The first line of thought, bioethical idealism, posits that payments and gifts are a means of showing respect and protecting participant autonomy in order to advance voluntary contributions to science and development. By this definition, payment is not coercion (Largent, Grady, Miller, & Wertheimer, 2013). Institutional review boards, universal ethical principles and highly-informed consent sessions are critical mechanisms towards preventing exploitation and unintended inducement (Dickert, Emanuel, & Grady, 2002; Grady, 2001).

The second line of thought, bioethical pragmatism, is generally sceptical of appeals to humanism, gift-giving, and altruism in clinical research. From this perspective, precarity and systematic inequalities are seen as key factors which contribute to the participation of healthy volunteers in clinical research (Bernstein, 2003; Chambers, 2001; Fisher, 2013; Lemmens & Elliott, 2001; Walker, Cottingham, & Fisher, 2018). As such, the engagement of healthy volunteers is understood as labour without labour protection (Cooper & Waldby, 2014; Parry, 2015). Contracting volunteers as “clinical labour” is seen as a radical way to protect participants.

Despite the diversity of nuanced arguments about the ethics of payments in research, there remains a tendency to discuss the role of benefit provision without consideration of the historical, political and social contexts in which research projects are carried out (Biruk, 2017). These locally existing forces are known to cumulatively influence outsourced research, shaping its local realities – what is documented as “ethical variability” (Petryna, 2005). Ethical variability is documented in numerous anthropological studies: ethnographies of trials that explore how assumptions about science, medicine and ethics interact with local practices and knowledge (Bull, Farsides, & Ayele, 2012; Folayan & Allman, 2011; Geissler, Kelly, Imoukhuede, & Pool, 2008; Gikonyo, Bejon, Marsh, & Molyneux, 2008; Hausmann Muela, Ribera, Mushi, & Tanner, 2002; Kingori, 2015; Lairumbi et al., 2008).

The notion of ethical variability can be useful to illuminate the dynamic tension between idealist and pragmatic justifications for paying healthy volunteers. Global health research usually takes place in settings where high levels of poverty, formal unemployment, and social vulnerability are common. In light of a growing interest in conducting empirical studies to inform bioethics, this paper examines participation in an Ebola vaccine trial in West Africa, documenting how healthy volunteers understood payments and participation in research in an Ebola and post-Ebola scenario, and how unique ethical, operational and socio-political challenges influenced the experiences of participants.

## Materials and methods

### The trial

In 2016, a phase II double-blinded placebo-controlled trial investigated the effects of two experimental Ebola vaccines against placebo in one of the countries in West Africa. We do not disclose the name of the trial and the location due to ethical reasons for protecting anonymity. The trial was organised as a collaboration between national and international health authorities, global health institutions, and the pharmaceutical industry, and became the first and the largest randomised clinical trial in the country. Trial organisers deployed complex community mobilisation procedures aimed at delivering participant-centred and culturally-sensitive interventions in local communities. The trial was successfully completed shortly after the end of the Ebola outbreak, boasting high enrolment and compliance rates.

### The study

This study is a qualitative single-site exploration of motivations, perceptions and experiences of volunteers who finished participation in this trial. Due to the retrospective design, we did not witness the original informed consent process for the clinical trial participation. This investigation takes a neutral stance, and was designed, conducted, and analysed independently of trial sponsors, organisers and community mobilisation teams. This study was inspired by the academic call for a more empirical bioethics (Bull et al., 2012; Dunn, Sheehan, Hope, & Parker, 2012), and a need for ethnographic exploration of Ebola-response practices with a focus on views from below (Ebola Response Anthropology Platform, 2014). Trial participants were interviewed in semi-structured interviews and focus-group discussions (FGDs). The question guide was adapted from the framework of so-called “consent studies” (Bull et al., 2012), and was further adjusted throughout data collection. The final version can be found as Appendix.

The project was formally approved by ethics boards of both the [host university] and the local university ethics board, and it was informally approved by local community leaders and researchers conducting the trial. Participants signed a written, informed consent form to participate in the study. Communication with informants was carried out in English (the official language), with the help of a local assistant proficient in vernacular English. The informed consent form contained an option for participants who agreed to be audio-recorded. Audio recordings were transcribed by the local assistant present during all data-collection sessions. The transcripts were then edited for clarity and consistency using MS Word. Transcripts were analysed using inductive thematic analysis, accompanying field notes and debriefing discussions. The Ebola vaccine research team was provided with preliminary results from this project.

### Roles of authors

Author A, is a trained bioethicist and a PhD student at [University] who is not affiliated with trial organisers and community mobilisation teams. They conducted interviews and focus groups with trial subjects in communities which hosted the trial. Author A was supported by a local assistant also independent from the Ebola vaccine research team. Author B was involved in the Ebola response as a physician and anthropologist with a number of trials in other Ebola-affected countries, and was familiar with the context in the country where author A conducted research. Author B supervised the work of author A and provided various important insights into preparation, conduct, analysis and interpretation of findings from this study.

## Informants and data collection

Author A collected data over three months of fieldwork in areas which hosted the trial (October –December 2016). At that time, the Ebola outbreak and the trial were officially completed. In total, 25 trial volunteers (referred throughout this text as “informants”), mostly men, who had completed participation in the Ebola vaccine trial, were interviewed in 11 semi-structured interviews and 5 focus group discussions. Aware of the peer stigma affiliated with the status of being a trial volunteer, snowball and respondent-driven sampling strategies were implemented, starting in locations around the research unit where the assistant knew that trial subjects frequented. Recruitment was guided by an initial group of informants who connected Author A and the assistant with other trial subjects. All informants were interviewed in the vicinity of the research unit (radius approximately 2 km). Places and times for data collection were selected by informants. Data was collected in public settings, so numerous people were present. All informants were legal adults living in states of deprivation, with limited access to food, safe drinking water, sanitation facilities, health, shelter and formal education, conditions in keeping with the area which hosted the trial. All informants reported an absence of stable income, and routinely combined various forms of flexible labour providing less than the national minimal wage. Data was collected at the time of rumours, fears and disappointment surrounding Ebola response and research. Several people terminated their participation due to the belief that researcher A was affiliated with trial organisers.

Nine of 11 interviews and five FGDs were audio-recorded. Approximately two-thirds of the study population said they were illiterate; the assistant explained study procedures in lay language. Interviews and focus groups lasted between 20 and 60 min, depending on the point of saturation. Participants were compensated with an equivalent of USD 2.50 as was required by the local ethics board. “Compensation” was of particular importance for individuals seeking payments, resulting in a high demand to be interviewed at the late stages of data collection. Group discussions and interviews produced various responses and were connected to the fact that Researcher A was perceived as a foreigner affiliated with the wealthy developmental apparatus, who, yet again, conducted “slum” research in locally troubled areas. Response patterns varied from emotional discussions led by participants themselves, mostly during FGDs, to more formal individual discussions which were perhaps shaped by the awkwardness of being interviewed by a foreigner of a higher social and economic class.

## Results

Five main themes which emerged from the discussions with informants about participation: hope for support, importance of payments, problems with follow-up, Graduation and ID cards, and unfulfilled expectations.

### Hope for health, training and support

They told us about the future benefit, if you take the vaccine, after the vaccine study you will get something good for yourself … future benefit that encouraged most of us to go there. (FGD 5)

Informants stated that they voluntarily accepted to participate because Ebola vaccines generated hopes of protection from the disease, they would be getting quick money to satisfy immediate needs, and because they were promised that their lifestyle would improve in the longer term. Informants explained that those interested in enrolling in the trial were asked to obtain appointment cards stating the date and time for “workshops”. They further explained that in the workshops study educators informed potential participants about the details of participation, follow-up, community engagement, payments and future benefits. According to informants, there were long lines to obtain the appointment cards. Due to the fact that many people wanted to participate, people sometimes had to queue several times in order to gain admission to the pre-consent workshop.

The long waiting periods facilitated identification of informants by those community members who held negative views about clinical research which led in some cases to their stigmatisation. Informants reported that at the time rumours were proliferating within communities that the Ebola virus was actually being spread by powerful Ebola vaccine researchers. While long waiting periods discouraged those worried about the prospect of being exposed to stigma and discrimination, it did little to discourage marginalised individuals because they were inured to stigma, as they experienced it on a daily basis. Some informants reported hiding the fact that they were participating for fear of losing their jobs or straining relations with family and friends. Most informants commented on the fact that their participation in the trial had been supported through a collaboration between the government and a northern research institution. Several informants described the workshops as an educational activity wherein people could study Ebola, aspects of clinical research and ways to be research volunteers, as one of informants concluded: “The workshop will educate you” (Interview 2).

The following quote exemplifies the importance of the workshop as a study platform provided by the research teams:

Interviewer: how was Ebola research introduced to your community?

Interviewee: anybody who interested to take [the vaccine], you can go there. Before going there, you got to study … you got to go for workshop … they can nurture you. (FGD1)

As numerous informants lived with limited access to food, packages of food during the workshop were of great significance for informants, as the following quote exemplifies:

Before you attend the workshop, they shared a package, in it there was biscuit and other snacks and they usher us in ten at a time. It was guarded by securities. (Interview 3)

Informants reported that they were provided with identity cards. One informant further explained the process:

Interviewer: Can you tell me about the workshop?

Interviewee: Before taking the vaccine, they take your picture and they issue you a card. During the workshop, they nurture you. They are not forcing you, it’s voluntary … They give you a package of food to eat. (FGD 2)

In the context of widespread unemployment, lack of formal education and lack of socioeconomic opportunities, expectations of long-lasting collaboration and prospects of future benefits were key reasons cited for participation, despite stigma against informants:

[Researchers] just told us that the USD 40 was just a little compensation but we had a better future ahead of us after the programme that we will tell our friends that hated us that we participated, and we are happy. (Interview 8)

### The role of payments

At that time, it was like war fighting. Because people were dying. It was more like a [civil war] crisis. So, people who went to take the vaccine they did it at their own risk because they wanted money to buy their pills to protect themselves … I wanted to survive, to get little cash. (FGD 2)

One informant showed the consent form (version 1.0 January 30, 2016) form which contained the statement, “you will get paid for your time after each study visit or contact”, without specifying the amount. Informants stated that the details about payment, such as frequency and values, were given verbally by the research staff, as this informant further explains:

In the workshop they explain and they told us how much we were going to be receiving for the vaccine. They just talk it in mouth. We didn't sign anything for it. It was just done in mouth. (Interview 1)

Informants consistently reported the following payment scheme: USD 20–40 for a vaccination visit and USD 10 for a follow-up visit. Participants informed that they were provided with an additional USD 150 in the end. Informants reported that an average monthly salary in the research area was about USD 60, a generous amount. For instance, for an equivalent of USD 16 it was possible to buy a 25 kg bag of rice, while USD 0.05 is enough to buy a pack of drinking water. A key emphasis was made on the dire socioeconomic situation in West Africa:

The most important information I heard about the vaccine was the future benefit they promised us … I bought food and some clothes because life here is hard. (Interview 8)

Informants used these payments for purposes such as buying goods, investing in microbusinesses, paying school fees, or fixing up rooms. They said that they used the benefits of participation in the same manner employment wages would have been used. In some cases, payments in research were the only income people had, and at least one informant participated not knowing about the purpose of the vaccine:

Interviewer: Can you please tell me why you decided to participate in the trial?

Interviewee: For me, I took the vaccine due to the money they were giving along with the vaccine because I wasn’t working at that time. It was later I knew that the vaccine was intended for prevention. (FGD 4)

### Problems with follow-up

If you skip the tracker visit you won’t graduate and won’t get the rest of your money. If the tracker doesn’t see you he won’t make a report on you so you won’t receive your money … because they had to keep record on you. (Interview 4)

Participants further explained that in pre-consent workshops they were informed about “trackers”, people with formal powers in communities who were employed by researchers to track participants outside the research unit. Compliance with researchers’ and community outreach workers’ demands was seen as an unspoken requirement for securing benefits. Some informants expressed concerns about upsetting researchers and subsequently being disqualified from the study. Informants developed an impression that it was essential to communicate with trackers to keep their affiliation with the trial, and reported a perceived absence of sufficient tracker communication and structural inabilities to deliver their concerns and complaints, an experience which contrasted with the image of caring trackers at workshops:

They give us a form including their phone number; but whenever we call the number indicated on the form, no response … They even took the ID cards that was given to us after we took the vaccine and give us a certificate. (FGD 3)

Some informants were afraid that if they withdrew from the trial, they would not be informed whether they had received the Ebola vaccine or a placebo:

They had an appointment paper, if you don’t go you won’t receive your money … if you don’t want to attend the appointment that means you are out of the programme … will lose the benefit, and we had to know the result from the vaccine we took. (Interview 9)

Of particular difficulty for informants was approaching trackers, due to their formal power in the community and to structural markers that divided marginalised participants from other community members:

Interviewer: Did you have any issues with participation?

Interviewee: Yes, my tracker was assigned to me to be checking up on me but to even see him was difficult. Trackers weren’t paying full attention to us, we only see them on the street. (FGD 2)

This perceived problem was raised by multiple informants, as the following quotes exemplify:

The trackers will tell you at the hospital to lie and tell the people that we used to meet. (Interview 3)

I have a problem with my tracker he is someone who never visits me, since I became a participant I always see him at the hospital. (Interview 5)

Some informants reported the pressure to share beneﬁts they received. In particular, some participants stated that they experienced pressure to kick back benefits to trackers and other community members:

Interviewer: Can you tell about the follow-up?

Interviewee: If trackers have problems with you, you will be disqualified … People who were talking bad words they asked us to share money with … even trackers … they wanted cold water … but [the] tracker benefited more than me, so I did not give him any money. (FGD 5)

“Cold water” is a colloquial English term for a small bribe for provided services, which refers to the 500 ml pack of water sold by street sellers. Because informants did not receive payments for trackers’ visits, the positive image of the tracker among participants was a person who provided his or her own money for participants. On the contrary, the following quote exemplifies the image of a good tracker:

The only efficient tracker, he used his own money to give it us, even to ones who he was not tracking … [USD 3-4] … encouraging me. (FGD 5)

Finally, some participants explained that food packages for volunteers became smaller alongside the timeline:

Interviewee: I want to ask you a question: do you think that it was right that they give us biscuits and juices to eat after we took the vaccine?

Interviewer: Yes

Interviewee: When they started firstly the biscuits they were offering us were much thicker than later. (FGD 3)

### Graduation and ID cards

The new [trial subjects] that just coming for the new study will be asking for idea for it, we going to tell them what we do … like a teacher, we graduated first. (FGD 5)

“Graduation” was what informants called the very last visit in the follow-up period, scheduled approximately six months after the initial vaccination visit. Informants explained that during the graduation visit, participants handed in their study ID cards, and in return, they received a certificate of participation and additional payments:

They just told us that they will give us USD 150 after we graduate and it was what they give us, and the risk was my life because I risk my life to take the vaccine. (Interview 6)

Informants showed their A4-sized, diploma-like certificate which was signed by three research team representatives and stated that the named recipient had completed his or her participation in 2016. Graduation certificates were perceived by informants as proof that they were responsible for research subjects who adhered faithfully to research protocols. Graduates highlighted their willingness to work with researchers to supervise newcomers, advertise vaccines or perform other procedures that might extend their formal affiliation with the researchers. Especially problematic for informants was the fact that participation – and the risks involved – did not lead to employment in the research sector as expected. Participants were frustrated by the invisible line that divided them from those employed by the trial, who had formal contracts and had ongoing privileges:

Interviewer: what do you mean by benefitting from graduation?

Interviewee: Benefiting like be the ones carrying awareness in those various counties … But people who did not take it are the ones working with them now. We already have the vaccine in us, and our benefit was to work along with them. Because as we're working along with them. We are the ones who know the effect of the vaccine. We are the ones who will tell other people whether or not it is good. They put us aside and they went on to bring their nieces from on the side and they started benefiting the money. (FGD 2)

Informants listed mobile phones and shirts with trial symbols among the missing benefits:

We should have even received shirts from them pretending to the vaccine, but they give the shirts out to some of their family members who were advertising the vaccine. (Interview 3)

Some informants expected that, through ID cards, they would be provided benefits beyond graduation. The common narrative was that researchers purposefully took the ID cards to receive the benefits to which participants were entitled:

I believe that any benefit that comes from the West they will used our name to benefit instead of us, so we requested for the ID cards to be in our position … the ID card should stay with me until I get old because I participated in the programme. Because there are other benefits they don’t want us to be the beneficiary instead they want their kids. (Interview 5)

Graduated participants were invited to the next Ebola vaccine trial and follow-up programmes. There was a wide range of perspectives regarding additional participation: for some informants, it was important that they could participate again; for others, the prospect of a new trial was seen as a false hope:

So, what I want to ask, if they bring the [new Ebola vaccine] will I take part in it again? (Interview 1)

### Unfulfilled expectations

[Participation] is like you're carrying someone else's child to the line and tell them say you're going to fight for me but if we capture this place you will be benefited, and these initial soldiers I took with me and get them sidelined and gave the benefit to another troop. How will I be looked at? I will be looked at ugly. (FGD 2)

The excerpt above exemplifies how one informant, also an ex-combatant, compared his experience in trial participation with the past civil war conflict. In doing so, he made a parallel between the ways in which civil war generals used child soldiers as a form of disposable power, and the ways in which researchers approach trial participants. After graduation, numerous participants described a return to unemployment with the additional burden of the stigma of having been a research participant. Informants also described disillusionment with research, characterised in terms of broken promises and missing items and benefits. For instance, participants expressed concerns that they had missed the opportunity to go to the USA for networking purposes:

[Researchers] said that they were going to carry us to different countries for people to see us because this vaccine is worldwide stuff and they want to get the result from us. And they lied to us and betray us. They said they were going to carry us to Washington DC. (FGD5)

For informants, the injustice lay in the fact that they were the ones who took a biomedical and societal risk associated with the trial, but unlike researchers, trackers and security guards, they were not entitled to post-graduation benefits. The following quotes exemplify this broad concern about an absence of “compensation” and a concern that the original “agreement” was not met:

They actually made an agreement with us; they said if we were going to be successful during the vaccination process we will benefit … We actually risk a lot and now we are not getting anything. (FGD 4)

Informants subsequently reported that their trust in researchers declined alongside the research timeline as they realised their initial expectations would not materialise:

Trust changed because what they told us change, they started reducing the money they promise us, so I told them we are taking a great risk and we were neglected by our family, and it’s something that can bring chaos. (Interview 8)

Informants stressed the inequality between different trial stakeholders, and the fact that clinical research in Africa is generally a less lucrative activity than when undertaken in the West. Many believed that the vaccine would be sold on the global market, bringing more benefits to researchers and other stakeholders. In the quote below, an informant expressed a deep frustration with the whole process:

USD 590 every month [for trackers] … Security guard is making USD 450 … . They ate our money. That is what getting them fat … Whereas in Europe if you take vaccine you can be benefited more than here. The people who did not take [the vaccine] where the ones who benefited. They brought the vaccine and if their vaccine is real they will put it on the global market. We know this. They will sell that vaccine that Ebola vaccine study … They are all criminals. (FGD 2)

## Discussion

### Between emergency, pragmatism and idealism

Significantly, results from this study show how responses to precarity emerged as a key motif in the narratives of informants about their voluntary participation in an Ebola vaccine trial in West Africa. Data collected suggests that informants’ experiences were shaped by long-term social and economic problems, memories of horror and violence during the earlier civil war, as well as the outbreak itself. Trial participation offered the fragile hope that the vaccine would prevent Ebola, despite rumours prevalent in participants’ communities that the vaccine trial itself was a source of the disease devastating West Africa. The trial, the first large randomised clinical trial in the country, was able to provide support to participants that would otherwise have been unavailable. The meaning of “participation” and benefit provision differed considerably from that of volunteerism, transport and wage compensation normally assumed in bioethical considerations. Making a virtue of dire necessity, the participation of volunteers in research had a dynamic survivalist character, with a simultaneous presence of factors influencing autonomy and heteronomy. As such, the process of voluntary decision-making was largely affected by macro-level factors, which embedded benefit provision in the context of constrained health, social and economic opportunities.

In particular, informants (i) saw “participation” as improvised employment while expressing dissatisfaction about being at the very bottom of the clinical research pay scale; (ii) expressed a willingness to remain in research and follow-up as long as possible because of the prospect of acquiring additional benefits; and (iii) considered their enrolment to be a risky exchange wherein required compliance was compensated with benefits. In accordance with clinical research practices, social support (in the form of payments, better access to health care and food packages) ended after the graduation once the active phase of the trial was over, generating dissatisfaction amongst the graduates. Top-down power dynamics between trackers, security guards, and informants shaped frustration and disappointment following graduation practices. A similar situation of post-trial disappointment was documented in Ivory Coast when participation in HIV research was understood as a platform for long-term reciprocity and a sign of virtuous activities like sharing and caring, which ended “unexpectedly” at the end of participation (Marcis, 2015). Our findings concur with previous ethnographic studies that show how normative styles of ethical reasoning risk misrepresenting motives to participate as altruism, masking internal frictions, tensions and considerations. This suggests that pragmatic considerations should inform policy and practice in global health research, as idealist justifications fail to capture pragmatic complexities which participants have to routinely overcome.

### Ethical variability: clinical research as an inadvertent reintegration programme

Ethical variability implies that the contexts in which a clinical trial is carried affect how research is locally perceived and carried out. An important analytic insight is that the experiences and perceptions of trial subjects might be linked to other processes that operate within similar social and historical contexts. A question thus emerges: what is the contextual meaning of a graduation from an intervention such as Ebola trial?

When Ebola struck, memories of civil war and, significantly, post-war reintegration and reconstruction programmes were still vivid in Liberia and Sierra Leone. In 2004-2005, the first wave of reintegration programmes – “Disarmament, Demobilisation and Reintegration” (DDR) – began with the goal of rehabilitating soldiers who had fought in the civil wars which ravaged Liberia and Sierra Leone between 1989 and 2003. The revised generation of reintegration programmes – “Reintegration, Rehabilitation and Recovery” (RRR) – was launched in 2005, targeting a broader spectrum of ex-combatants and non-combatant community members alike (McMullin, 2013). Despite the diversity of reintegration programmes, they shared a goal of establishing a socioeconomic mechanism for intended beneficiaries to get stable employment and income through participation in organised activities. Graduation ceremonies and training certificates were provided for participants who completed the programmes (Munive & Jakobsen, 2012). Numerous DDR/RRR graduates experienced re-marginalisation and disappointment once the programmes officially ended (Bøås & Bjørkhaug, 2010; Kilroy, 2014; Maclay & Ozerdem, 2010; McMullin, 2013; Munive & Jakobsen, 2012), similar to post-graduation narratives we encountered in this study.

Data collected further suggests striking similarities between reintegration programmes and Ebola vaccine research in how participants understood the purpose of study ID cards. Our informants saw ID cards as proof of a “research citizenship” securing benefits the state was not able to provide, which were taken away after graduation, leaving participants frustrated. Kilroy described the identical understanding of ID cards among graduates from reintegration programmes: “the experience with the identity cards left many ex-combatants with the impression that their beneﬁts were being siphoned off by people working in the agencies” (Kilroy, 2015). Moreover, common to both reintegration programmes and Ebola vaccine research were pre-participatory aspirations of participants; ongoing, temporary social protection during participation; and unmet expectations after graduation, which appeared in stark contrast with the perceived wealth and power absorbed by the implementers (Kilroy, 2014).

This parallel reflects a historical reality: numerous contemporary projects on socioeconomic and political development directly and indirectly absorbed the infrastructure of reintegration programmes. The important examples include the “Youth, Employment, Skills” project launched by the World Bank (2010–2016), “Quick Impact Projects” organised by United Nations Mission in Liberia (2017–2020), as well as various NGOs replying to the call of the Ministry of Labour for job creation and youth empowerment (2015). Our data suggests that contemporary Ebola trials were influenced by the legacy of these prior participatory interventions that addressed rather different kinds of problems. Similar to reintegration programmes, clinical research generated hopes of employment after participation. Likewise, a graduation certificate was perceived to give an advantage when entering a new job market in the assumed post-Ebola expansion of the health care systems. Informants’ most optimistic expectations were to participate as human subjects in trials offering higher pay, or to become members of research teams holding formal employment contracts. However, graduates of reintegration programmes in many cases were unable to secure employment after graduation, which led to their marginalisation instead of reintegration (Bøås & Bjørkhaug, 2010). When similar practices are used by different programmes and institutions to different ends, their more recent iteration is often interpreted in light of prior experiences and discourses; older meanings persist, constituting a form of symbolic misattribution. This seems to have occurred in the case of the Ebola trials, where participation in reintegration programmes’ similar design led participants to expect similar outcomes.

### Participation in interventions as precarious labour

Can participation in interventions such as reintegration programmes or clinical research be conceptualised as labour for obtaining valued health, social, and economic benefits? And, if so, how would this affect research ethics in practice and in theory?

The understanding of labour is often represented in popular discourse as a morally positive activity combining wage economy, formal contracts, and a fixed workplace. However, in the era of flexible accumulation, labour markets are being fundamentally restructured, promoting precarious work regimes in a less regulated fashion (Harvey, 1991). Labour in many African contexts escapes a dogmatic coupling with the process of production, consisting of attempts to secure transfers of benefits from those who have them, to those who do not (Ferguson, 2015). In recent discussions, emerging forms of flexible labour are seen as evidence of a new globally emerging class of workers – the precariat, distinctively known to suffer from a lack of professional connections, insecurities about the future, and an absence of social protection and stable income (Standing, 2014). By navigating between a large unemployment pool and limited employment options, the precariat becomes a disposable and readily-available force in proliferating labour markets in the Global South (Munck, 2013; Scully, 2016). In line with this, existing literature suggests that the paradigm of global health, being historically, politically, and practically connected to international development, is inherently surrounded by local pools of vulnerable workers seeking affiliations with powerful international institutions (Brown & Prince, 2016; Kingori & Gerrets, 2016).

These concerns are directly applicable to the context of West Africa as the Ebola outbreak worsened the socioeconomic situation across the region, exacerbating already fragile economies and depleting labour markets (Bowles, Hjort, Melvin, & Werker, 2016). As a result, numerous West Africans were put in positions of seeking new opportunities for financial survival for themselves and their families. In this sense, our findings align with those of Abadie (2015) who described the process of participation in clinical research in the US as an opportunity to temporarily or permanently overcome precarity, leading to “professionalisation” of research subjects. What remains a core question for research ethics is whether or not participation in clinical research should be regulated as a form of labour, as is suggested by pragmatic bioethicists (Cooper & Waldby, 2014; Parry, 2015). At this point, it is essential to note that the strength of contractualisation depends on the existence of a well-organised regulatory system and its availability to protect contract workers. This is unlikely in West Africa, where state capacity is limited and extreme poverty is widespread. Formal unemployment varies from country to country and remains generally high among youth. From an anthropological perspective, Geissler (2011) warned that an analytically convincing idea of contractualisation of research participation risks generating economic reductionism by labelling all participatory reasons as employment. In light of this debate, data from this study suggests that contractualisation of trial participation may provide a temporary reprieve, yet how much does this really change the structural conditions that promote precarity among a sizeable proportion of the population?

## Limitations

These site-specific accounts documented views of marginalised participants in 2016 shortly after Graduation, and these may have subsequently changed. A potential bias of this study is that the opportunity it provided participants to discuss problems with their research participation allowed painful individual experiences, as well as chronic societal forms of suffering, to be expressed. Such perceptions and do not necessarily represent the voices of other participants in this and other Ebola clinical trials in West Africa.

## Conclusion

This paper documented participants’ narratives on their experiences and perspectives in a unique historical event: an Ebola vaccine trial successfully implemented in exceptionally challenging settings in a West African context. In these settings, research teams strove to deliver the trial and protect the voluntary decision-making of participants by implementing good research practices and international guidelines. Simultaneously, trial volunteers approached participation as a rare health care and socioeconomic opportunity to be affiliated with powerful structures which would otherwise remain inaccessible. Sharp narratives of deep dissatisfaction among graduated participants were fuelled by perceptions of internal corruption, and reflected systematic inequalities and the overall failure of reintegration programmes. Despite adhering to ethical safeguards, prevailing forms of vulnerability at times meant trial participation could exacerbate and mitigate the vulnerability.

Similarities in design, perceptions and expectations between reintegration programmes (DDR and RRR) and the participatory component of Ebola clinical research suggest that addressing local context includes the history of prior interventions which can shape perceptions and expectations. For instance, “participation” was often perceived as a euphemism for exploitation. Due to the fact that the protection of human subjects remains the key priority for international research ethics, there is a great need to organise new forms of collective action and representation for participants approaching participation in interventions as a form of employment to meet basic needs in precarious settings. Further research in empirical bioethics is needed to understand the local meaning of “participation” in research in West Africa and elsewhere.

## Appendix

1. Can you please tell me a bit about yourself and what happened to you during the Ebola outbreak?
2. Can you please tell me how and when you decided to participate in the trial?
  Did you discuss your participation with family or/and friends?
  • Who made the decision?
  • Did you discuss your participation with somebody outside the compound?
  • Why did you participate, what was important for you?
  • Would it have been hard to cancel participation?
3. What was most important thing to know about vaccination before you went to the unit?
4. Can you please tell me what happened when you went to the unit?
  - Was it easy or difficult to sign up for participation?
  - Had you met fieldworkers at the unit before?
  - Did you feel comfortable with them?
  - Did you understand them easily?
  - What did they tell you about the vaccines?
  - Can you tell me a bit about the study ID cards?
  - Can you please tell me what happened during informed consent?
  - What was happening at the unit (busy/quiet?)
  - Did you feel comfortable asking questions or asking questions to be repeated?
  - Were you happy with the answers?
  - What did they tell you about the programme?
  - Was there anything you did not expect to happen?
5. What did the researchers tell you about the risks and benefits of your participation?
  - To you personally, what were the risks and benefits of your participation?
6. Were you provided with money or other things for your participation?
  - What did they tell you the money was for?
  - Can you please tell me how you used this money?
  - When did they provide the money during the visit?
  - Did they provide the same sum for everyone?
  - Did they tell you how this sum was chosen?
  - Did you feel you had obligations because of this money?
  - What else did you receive or were told you would receive in the future?
7. What did they tell you about the vaccine?
  - Please tell me how you felt when you returned from the compound
  - Did you feel protected after you received the shot?
  - Do you still think you are protected? If yes, against what diseases?
  - If yes, did it affect your everyday precautions and safety measures?
  - When did you find out what shot you had received (A/B/C)?
8. Can you please tell me how your participation affected your everyday life?
  - Did your fellow community members know you participated?
  - Did you experience any stigma because of your participation?
  - Did you know that your participation might bring stigma before you consented to participate?
  - Was possible stigma discussed during informed consent?
  - Who was fighting stigma and how was it done?
9. Can you please tell me a bit about follow up visits?
  - Who are trackers?
  - What happened during each visit?
  - Can you please tell me about the tracker and how you communicated?
  - Was the tracker available for you to ask questions, express concerns or complaints?
  - How many follow up visits did you have and who decided when to have them?
  - How much money was given per visit? What was this money for?
  - Did you think about cancelling or extending your follow up visits?
  - What kept you as a participant?
  - Other participants talk about the “Graduation”. Have you heard about it?
10. When you decided to participate, did you trust the researchers?
  - Did your trust change overtime?
  - What would be your advice for researchers and potential participants?
11. Is there anything we didn't cover that you wanted to discuss?
